# Semi-Quantitative and Quantitative [^18^F]FDG-PET/CT Indices for Diagnosing Large Vessel Vasculitis: A Critical Review

**DOI:** 10.3390/diagnostics11122355

**Published:** 2021-12-14

**Authors:** Olivier Gheysens, François Jamar, Andor W. J. M. Glaudemans, Halil Yildiz, Kornelis S. M. van der Geest

**Affiliations:** 1 Department of Nuclear Medicine, Cliniques Universitaires Saint-Luc and Institute of Clinical and Experimental Research (IREC), Université Catholique de Louvain (UCLouvain), Avenue Hippocrate 10, B-1200 Brussels, Belgium; francois.jamar@saintluc.uclouvain.be; 2 Department of Nuclear Medicine and Molecular Imaging, University of Groningen, University Medical Center Groningen, Hanzeplein 1, 9700 RB Groningen, The Netherlands; a.w.j.m.glaudemans@umcg.nl; 3 Department of Internal Medicine and Infectious Diseases, Cliniques Universitaires Saint-Luc and Institute of Clinical and Experimental Research (IREC), Université Catholique de Louvain (UCLouvain), Avenue Hippocrate 10, B-1200 Brussels, Belgium; halil.yildiz@saintluc.uclouvain.be; 4 Department of Rheumatology and Clinical Immunology, University of Groningen, University Medical Center Groningen, Hanzeplein 1, 9700 RB Groningen, The Netherlands; k.s.m.van.der.geest@umcg.nl

**Keywords:** [^18^F]FDG-PET/CT, giant cell arteritis, Takayasu’s arteritis, SUV

## Abstract

To confirm the diagnosis of large vessel vasculitis (LVV) with high accuracy, one of the recommended imaging techniques is [^18^F]Fluoro-2-deoxy-d-glucose positron emission tomography with computed tomography ([^18^F]FDG-PET/CT). Visual assessment of [^18^F]FDG uptake in the arterial wall compared to liver uptake is the mainstay for diagnosing LVV in routine clinical practice. To date, there is no consensus on the preferred semi-quantitative or quantitative parameter for diagnosing LVV. The aim of this review is to critically update the knowledge on the available evidence of semi-quantitative and quantitative [^18^F]FDG uptake parameters for diagnosing LVV and to provide future directions for methodological standardization and research.

## 1. Introduction

### 1.1. The Clinical Spectrum

Large vessel vasculitis (LVV) is a generic term that encompasses a heterogeneous group of disorders characterized by inflammation of blood vessels of large and medium-sized caliber. The main forms are giant cell arteritis (GCA, sometimes referred to as temporal arteritis or Horton’s disease) and Takayasu’s arteritis (TAK). Isolated, noninfectious aortitis can be added to the spectrum but has a different clinical picture [1].

GCA and TAK are systemic vasculitides characterized by mononuclear and granulomatous infiltration of the vessel wall [2,3]. The clinical picture, though, is quite different: GCA is more frequent among Caucasians and occurs mainly over the age of 50. It can affect the large systemic arteries (i.e., large vessel GCA; lvGCA) and medium-sized cranial arteries (i.e., cranial GCA; cGCA). In view of this, the associated morbidity can be highly significant, extending from permanent blindness and stroke in cGCA to aortic aneurysm dissection in lvGCA [4]. TAK is found mainly in female patients younger than 40 years, with a predilection for Asian origin. TAK seems to differ from GCA by the extent and localization of vessel involvement, which involves more widely the mesenteric, renal and iliofemoral arteries, whilst mostly preserving the medium-sized cranial arteries [5].

The diagnosis of these disorders is principally established by the combination of clinical symptoms and laboratory parameters [5,6]. This includes (i) nonspecific constitutional symptoms such as malaise, fatigue, fever, weight loss, (ii) localized symptoms such as headache, and jaw claudication in GCA, and limb claudication and loss of pulse in TAK, and (iii) laboratory parameters of nonspecific inflammation, including a raised C-reactive protein (CRP) and erythrocyte sedimentation rate (ESR), as well as inflammation-related anemia and thrombocythemia [7]. In addition, GCA may also occur together with polymyalgia rheumatica (PMR), with both diseases being described as a continuum, mostly in elderly patients [8].

The clinical symptoms and laboratory markers in GCA, TAK and isolated noninfectious aortitis are quite similar. The key issue is to make the correct diagnosis and to prevent damage by early and adequate treatment of vascular inflammation. High-dose glucocorticoids have remained the cornerstone of treatment in GCA and TAK. In addition, early addition of immunosuppressive treatments is advised in TAK, whilst this is also done increasingly in GCA [9].

### 1.2. The Role of Molecular Imaging

More than twenty years after the first description of a positive [^18^F]Fluoro-2-deoxy-D-glucose positron emission tomography ([^18^F]FDG-PET) in patients with aortitis [10], the technique has been widely used for establishing the diagnosis of large vessel vasculitis (LVV), as well as for assessing treatment efficacy in the regular clinical setting and in clinical trials. According to the EULAR 2018 recommendations, [^18^F]FDG-PET/CT may be used as a first-line imaging technique to establish the diagnosis of lvGCA [11]. It also has the advantage of revealing inflammation in peri-articular and extra-articular structures in the case of polymyalgia rheumatica (PMR) and can help to exclude underlying malignant diseases or infections that may mimic the clinical symptoms of LVV. The uptake of [^18^F]FDG depends on the expression of glucose transporters on various cell types, including acute and chronic inflammatory cells, endothelial cells or fibroblasts [12,13,14], but also on many tumor cells. As such, [^18^F]FDG uptake cannot be deemed specific of systemic vascular inflammation but can also reflect tumoral infiltration, atheroma-related inflammation and inflammation associated with vascular infections. However, patterns of metabolic uptake may hint towards one or the other condition. Furthermore, the accuracy of [^18^F]FDG-PET/CT in vasculitis is rapidly reduced after the initiation of glucocorticoid treatment [15,16].

Many studies proposed a variety of methods for interpreting [^18^F]FDG-PET exams in this clinical indication. All those methods are based on the intensity of the glucose analog uptake going from intuitive visual assessment to structured visual scoring and finally semi-quantitative evaluation. Visual indices such as the 4-point scale (ranging from 0 to 3) [10] and visual grading score (vessel uptake compared to liver background) [17] have been proposed, and the latter is currently recommended by the European Society of Nuclear Medicine (EANM) and the Society of Nuclear Medicine and Molecular Imaging (SNMMI) [18]. In addition, a visual-based composite score, known as total vascular score (TVS), evaluating uptake in 7 to 11 different vascular regions, has been developed for diagnosing LVV [18,19]. Although vasculitides and scoring methods for interpretation were first explored using [^18^F]FDG-PET, [^18^F]FDG-PET/CT has now become the standard in modern imaging, allowing for the development of semi-quantitative or quantitative approaches, such as aorta to liver SUVmax ratio, vascular/liver ratio, vascular/lung ratio and arterial/venous ratio [20,21,22]. Nevertheless, there is a lack of a standardized definition. The objective of this review is to critically update the knowledge on semi-quantitative or quantitative methods of [^18^F]FDG-PET/CT evaluation in the diagnosis and differentiation of LVV.

## 2. Methodology

### 2.1. Search Strategy

A comprehensive literature search of records through PubMed/MEDLINE databases was carried out until 5 August 2021. The following search algorithm combining several mesh terms was created and used: “large vessel vasculit * or giant cell arteritis or temporal arteritis or Horton or Takayasu arteritis or Takayasu AND fluorodeoxyglucose F18 or fluorodeoxyglucose or FDG or positron-emission tomography or positron emission tomography computed tomography or positron emission tomograph * or positron-emission tomograph * or PET AND sensitivity or specificity or sensitive * or specificit * or accura * or SUV or semiquant * or semi-quant * or TBR”. The search was restricted to English-language articles. No other restrictions were applied to the database search.

### 2.2. Study Selection

Titles and abstracts of the records were independently screened by two reviewers (O.G., K.S.M.G.) based on predefined inclusion and exclusion criteria. Inclusion criteria were original articles reporting information on the diagnostic accuracy (i.e., both sensitivity and specificity) of semi-quantitative PET-derived parameters for a diagnosis of GCA or TAK. Exclusion criteria were: (a) reviews, editorials, comments, letters or study protocols related to the review question; (b) case reports or case series (less than 10 patients included) related to the review question; (c) articles outside the field of interest of this review; and (d) articles not available in English. The full text of the selected articles was independently evaluated for its inclusion, and those who did not provide sufficient data with regard to the review scope were excluded from this review.

### 2.3. Data Extraction

Two reviewers (O.G., K.S.M.G.) collected and cross-checked information about studies and patient characteristics, including authors, year of publication, country, study design, patient population, number of patients, ongoing glucocorticoid treatment, presence of control group, the reference standard for LVV diagnosis, (semi-)quantitative index used and diagnostic performance parameters. Technical details on [^18^F]FDG-PET/CT imaging were not collected, but all included studies reported PET metrics corrected for attenuation.

## 3. Results

### 3.1. Literature Search

The comprehensive electronic database search of PubMed/MEDLINE identified 225 records, with the oldest reference dating from 2001. Overall, 213 records were excluded after title/abstract screening and full-text evaluation since those provided no information on the review question.

Twelve articles were eligible for a detailed description of the performance of (semi-) quantitative PET-derived metrics for diagnosing GCA (cGCA and/or lvGCA) and/or TAK.

### 3.2. Study and Patient Characteristics

Table 1 summarizes the patient characteristics and main results of the included studies [15,22,23,24,25,26,27,28,29,30,31,32]. All selected articles were published in the past decade. All studies except one were performed in Europe, and 75% (9/12) studies were retrospective in nature. All studies included GCA patients, while only two studies explicitly mentioned the inclusion of patients with TAK. More than half of the studies (7/12) also included at least some patients who received immunosuppressive treatment (mainly glucocorticoids) at the time of [^18^F]FDG-PET/CT. Different reference standards were used among the studies to establish the diagnosis of LVV, with 8/12 studies including temporal artery biopsy for a proportion of all patients. The ACR 1990 criteria [7] were used in five studies, while the diagnosis for at least some patients was made on clinical endpoints in three studies. One study did not specify the diagnostic criterion or reference standard.

### 3.3. Overview of (Semi-)Quantitative Parameters

The different (semi-)quantitative indices used across the included studies are listed in Table 1. Most of the studies (7/12) reported a target to background ratio (TBR). The reference organ for the TBR was the SUVmean or SUVmax of the liver (n = 6 studies), SUVmean or SUVmax of the vascular blood pool (n = 3 studies) and/or SUVmax of the lung (n = 1 study). A direct comparison of different reference organs was performed in two studies only. One study with non-treated GCA patients suggested that the liver as a reference organ provides better diagnostic accuracy than the blood pool [29]. Another study, in which some of the patients were already receiving treatment, suggested that the vascular blood pool might be better than the liver [22]. The diagnostic accuracy of the arterial SUVmean or SUVmax by itself, i.e., in the absence of a reference organ, was evaluated in 6/12 studies. Four studies used the SUVmax of the aorta wall with cut-off values ranging from 1.70 to 2.75 with large ranges in sensitivity and specificity, and with lower cut-off values usually yielding an increased sensitivity but decreased specificity. One study applied the SUVmax of the most active cranial artery with a cut-off > 5.0, resulting in a sensitivity of 79% and specificity of 92% for diagnosing cGCA [24]. Two studies indicated that a TBR provides higher diagnostic accuracy than a plain arterial SUVmean or SUVmax [29,30]. Three studies reported a total vascular score (TVS) which is a composite score of visual assessment of predefined vascular territories ranging from 7 to 11 vascular segments [15,26,28]. Optimal cut-off values differed significantly across these studies, with reported sensitivities and specificities ranging from 71% to 84% and 64% to 94%, respectively.

Only six studies, comprising 115 patients in total, performed a head-to-head comparison between visual interpretation (including TVS) and semi-quantitative indices [23,24,26,28,29,32]. In these studies, 148 subjects were considered as controls, mostly random oncology patients. No difference was found between the diagnostic performance of visual and semi-quantitative assessment (when using only the best performing semi-quantitative index per study, to avoid biases from unequal datasets) when using Youden indices (i.e., [sensitivity + specificity] − 1) for these six studies. The mean (s.d.) Youden index was 0.64 (0.14) vs. 0.67 (0.19) for the visual index and various semi-quantitative indices, respectively. Stellingwerff et al. confirmed by separately analyzing patients with or without glucocorticoid treatment that the diagnostic accuracy of visual interpretation and the TBR semi-quantitative indices was lower in patients on glucocorticoids, whilst no significant difference was observed for the SUVmax aorta [29].

## 4. Discussion

From this review, there is little information that guides the diagnosis of large vessel vasculitis towards semi-quantitative indices. Six studies allowed comparing the diagnostic performance of semi-quantitative versus visual indices in the same patient population. Since the diagnostic performance of a visual or metrical approach is always a balance between sensitivity and specificity, we evaluated the Youden index in these studies, which did not show any difference between both methods.

There may, however, be particular situations in which, on due knowledge, the interpretation of [^18^F]FDG-PET/CT may be mitigated. This is especially the case in elderly patients in whom uptake in atherosclerotic plaques may be observed, leading to a reduced specificity in identifying LVV. Even though an overlap exists and differentiating active vasculitis from atherosclerosis can be challenging, several PET uptake characteristics may hint towards one condition. Vasculitis typically appears as a linear, diffuse and circumferential uptake, while atherosclerosis rather presents as a patchy uptake with lower intensity. This does not hold true in younger patients with a suspicion of TAK but remains a diagnostic challenge in older and especially elderly patients in whom GCA can be suspected, as has been shown by Besson et al. [22].

From the data collected herein, there is no evidence that semi-quantitative [^18^F]FDG-PET/CT metrics may help to better diagnose LVV than visual scoring. Visual analysis or a qualitative metric based on visual analysis such as the TVS should remain the privileged way to make due interpretation of the so-called routine [^18^F]FDG-PET/CT. A similar conclusion was already made by Puppo et al. in 2014 [33] in a review on [^18^F]FDG-PET in vasculitis where stand-alone PET studies were included, whereas the current review focuses on hybrid PET/CT studies. In addition, the visual analysis showed very good interobserver reproducibility, which is essential for daily clinical practice, where the actual report is used for clinical decision-making [34]. Moreover, quantitative strategies usually require a certain experience and rigorous application of the methodology, e.g., drawing regions or volumes of interest on the arterial segments, which is time-consuming, operator dependent and not feasible in routine clinical practice. Another compelling reason to refrain from quantitative metrics such as SUV-based methods in clinical routine is related to the inappropriate application of literature-derived cut-off values for sensitivity and specificity in establishing the diagnosis of LVV. Indeed, a quantitative metric can only be correctly used/extrapolated to its own clinical practice if imaging characteristics affecting the metric such as voxel size, filtering, etc., are harmonized and standardized across different PET cameras. Arterial wall thicknesses are most generally in the range of 2–5 mm, and this raises a major issue of partial volume effect. This has not been addressed in LVV but was shown as a significant hurdle for assessing semi-quantitative data on vascular [^18^F]FDG uptake in the field of atherosclerosis [35]. Such impact in assessing large and medium-sized vessel activity in LVV is most likely similar, i.e., thresholds for pathological results are often exceeded in normal volunteers. Even though efforts have been made by publishing joint procedural recommendations, e.g., adherence to EANM Research GmbH (EARL) [18], achieving the highest level of standardization remains utopic at this time.

This patient population is at risk of sometimes severe and dramatic complications such as stroke or blindness. Therefore it is not uncommon that glucocorticoid therapy has been initiated before [^18^F]FDG-PET/CT will be performed. Nielsen et al. demonstrated that a few days on oral glucocorticoids did not change the diagnostic performance of the test, but that further on, the sensitivity decreased for visual interpretation [16]. In a sub-analysis of one report [29], the authors demonstrated that the diagnostic accuracy of TBR-based semi-quantitative indices was also reduced in patients under glucocorticoids, underscoring that [^18^F]FDG-PET/CT exams in patients on glucocorticoid treatment should be interpreted with caution. Only the SUVmax aorta showed better accuracy in patients with glucocorticoid treatment than in those without.

Nevertheless, semi-quantitative or quantitative indices may have an added and complementary role in clinical research trials. In these trials, consistency and congruency of imaging characteristics are of utmost importance since patients are often imaged at different time points or during treatment. Indeed, a recent systematic review suggested that TBR-based semi-quantitative indices might potentially be more responsive to change than composite scores based on visual assessment [36]. The best parameter to be applied in clinical studies is yet to be determined, and well-designed clinical trials may provide an answer to the value and indication of quantitative PET in LVV.

## 5. Conclusions

There is currently no evidence that semi-quantitative indices surpass visual analysis of [^18^F]FDG-PET/CT for diagnosing LVV. Visual analysis should remain the standard of care by looking at vascular distribution, depending on the suspected disease and the intensity of uptake as compared with general vascular or, as a surrogate, liver activity. Semi-quantitative indices, based on ratios, shall be implemented in clinical trials only with a well-defined calculation. In the future, large multicentric studies should focus on determining ONE single parameter that can be used in all studies and could further be implemented in clinical practice. The no-go of beyond three days glucocorticoid treatment should be enforced in all such studies before data can be used truthfully in daily clinical practice.

## Figures and Tables

**Table 1:** **Overview of studies reporting diagnostic accuracy of semi-quantitative indices for GCA or TAK**. Only studies in which all patients underwent [^18^F]FDG-PET/CT were selected. † At least part of patients receiving treatment at the time of the scan. †† At least part of the patients received treatment for less than 3 days before the scan. FUO = fever of unknown origin. GCA = giant cell arteritis. ICV = inferior caval vein. IJV = internal jugular vein. SCV = superior caval vein. TAB = temporal artery biopsy. TAK = Takayasu’s arteritis. TVS = total vascular score.

Authors and Year	Country	Study Design	Patient Population	No. of Patients	Control Group Yes/No	No. of Controls	Reference Standard LVV	(Semi-) Quantitative Index	Sensitivity	Specificity
Rottenburger et al. 2021	Switzerland	Retrospective	Suspected GCA †	17 (GCA)	Yes (suspected GCA but final diagnosis not GCA)	17	GiACTA trial criteria	Visual FDG uptake at temporal artery (visible)	53%	100%
								TBR: SUVmax temporal artery/SUVmean liver > 1	53%	100%
Nienhuis et al. 2020	Netherlands	Retrospective	cGCA ††	24	Yes (oncology)	24	TAB positive	Visual > background	83%	75%
								Visual ≥ liver	58%	96%
								SUVmax > 5.0	79%	92%
Imfeld et al. 2019	Switzerland	Retrospective	Suspected GCA †	68 (GCA)	Yes (suspected GCA but final diagnosis not -GCA)	34	TAB or ACR 1990 or incomplete ACR 1990 with positive imaging findings	TBR: SUVmax supra-aortic arteries/SUVmean liver > 1 and/or TBR: SUVmax aorta or iliofemoral arteries/SUVmean liver >1.3	72%	85%
Vaidyanathan et al. 2018	United Kingdom	Retrospective	Suspected LVV	17 (LVV)	Yes (suspected LVV but final diagnosis not LVV)	19	Clinical diagnosis	TVS (range 0–21) > 3.5	79%	94%
								TBR: SUVmax aorta/SUVmax liver > 1.0	76%	100%
Imfeld et al. 2017	Switzerland	Retrospective	Suspected GCA †	68 (GCA)	Yes (suspected GCA but final diagnosis not GCA)	35	TAB or ACR 1990 or incomplete ACR 1990 with positive imaging findings	TBR: SUVmax supra-aortic arteries/SUVmean liver > 1.0	71%	91%
								TBR: SUVmax thoracic aorta/SUVmean liver > 1.3	25%	91%
								TBR: SUVmax abdominal aorta/SUVmean liver > 1.3	34%	91%
								TBR: SUVmax iliofemoral arteries/SUVmean liver > 1.14	26%	91%
Clifford et al. 2017	Canada	Prospective	New-onset GCA †	28	Yes (oncology)	28	ACR 1990 and clinical diagnosis	Visual TVS (range 0–24) ≥ 9	71%	64%
Castellani et al. 2016	Italy	Retrospective	New-onset GCA/TAK	25	Yes (oncology)	15	Unclear	Visual TVS (range 0–33) > 8	84%	87%
								Average SUVmean > 0.697	96%	87%
Stellingwerff et al. 2015	Netherlands	Retrospective	New-onset GCA	12	Yes (inflammation, atherosclerosis, normal)	53	ACR 1990 or TAB or clinical diagnosis	Visual	92%	91% *
								SUVmax aorta > 2.75	83%	62%
								TBR: SUVmax aorta/liver > 1.03	92%	92%
								TBR: SUVmax aorta/SCV > 1.48	92%	73%
								TBR: SUVmax aorta/ICV > 1.72	75%	96%
Martinez-Rodriguez et al. 2014	Spain	Prospective	Suspected LVV	25 (LVV)	Yes (oncology)	15	Clinical and biochemical data, treatment response; TAB (n = 6) and MRA (n = 2)	SUVmax aorta wall > 1.74	80%	83%
								TBR: SUVmax aorta wall/lumen > 1.34	100%	94%
Prieto-Gonzalez et al. 2014	Spain	Prospective	New-onset GCA †	32	Yes (oncology)	20	TAB	Mean SUVmax > 1.89	80%	79%
								Mean SUVmax supra-aortic > 1.70	81%	79%
								MeanSUVmax aorta > 2.25	90%	42%
								MeanSUVmax aorta > 2.65	58%	90%
Besson et al. 2014	France	Retrospective	GCA †	11	Yes (infection, oncology)	11	TAB	TBR: highest SUVmax aortic arch/highest SUVmax liver > 0.71	82%	55%
								TBR: average SUVmax aortic arch/average SUVmax liver > 0.83	64%	82%
								TBR: highest SUVmax aortic arch/SUVmax lung > 7.46	82%	73%
								TBR: average SUVmax aortic arch/SUVmax lung > 6.9	73%	73%
								TBR: highest SUVmax aortic arch/highest venous blood (Right IJV) > 1.53	82%	91%
								TBR: average SUVmax aortic arch/average venous blood (Right IJV) > 1.63	82%	73%
Lehmann et al. 2011	Germany	Retrospective	GCA or TAK	20 (GCA, n=17; TAK, n = 3)	Yes (oncology)	20	ACR 1990 or histology	Overall visual interpretation	65% (data for 13 patients and 16 controls only)	80%
								SUVmax > 1.78	90%	45%

*: average of the three subgroups of controls (specificity: 94%, 79%, 100% in arterial inflammation, atherosclerosis and normals, respectively).

## Data Availability

Not applicable.
